# Prognostic value of circulating tumor cells associated with white blood cells in solid cancer: a systematic review and meta-analysis of 1471 patients with solid tumors

**DOI:** 10.1186/s12885-023-11711-7

**Published:** 2023-12-12

**Authors:** Mingguang Ju, Ziming Gao, Gaoxiang Gu, Haibo Huang, Anqi Sun, Chen Zheng, He Li, Yixiao Zhang, Kai Li

**Affiliations:** 1 Department of Surgical Oncology and General Surgery, Key Laboratory of Precision Diagnosis and Treatment of Gastrointestinal Tumors, Ministry of Education, Heping District, The First Affiliated Hospital of China Medical University, 155 North Nanjing Street, Shenyang City, 110001 China; 2 VIP International Department, Heping District, The First Affiliated Hospital of China Medical University, 155 North Nanjing Street, Shenyang City, 110001 China; 3 Department of Anesthesiology, Heping District, The First Affiliated Hospital of China Medical University, 155 North Nanjing Street, Shenyang City, 110001 China; 4 Department of Ultrasound, Heping District, The First Affiliated Hospital of China Medical University, 155 North Nanjing Street, Shenyang City, 110001 China

**Keywords:** Circulating tumor cell-associated white blood cells, Circulating tumor cell, Solid tumors, Metastasis, Prognosis

## Abstract

**Background:**

The clinical relevance of circulating tumor cell-white blood cell (CTC-WBC) clusters in cancer prognosis is a subject of ongoing debate. This study aims to unravel their contentious predictive value for patient outcomes.

**Methods:**

We conducted a comprehensive literature search of PubMed, Embase, and Cochrane Library up to December 2022. Eligible studies that reported survival outcomes and examined the presence of CTC-WBC clusters in solid tumor patients were included. Hazard ratios (HR) were pooled to assess the association between CTC-WBC clusters and overall survival (OS), as well as progression-free survival (PFS)/disease-free survival (DFS)/metastasis-free survival (MFS)/recurrence-free survival (RFS). Subgroup analyses were performed based on sampling time, treatment method, detection method, detection system, and cancer type.

**Results:**

A total of 1471 patients from 10 studies were included in this meta-analysis. The presence of CTC-WBCs was assessed as a prognostic factor for overall survival and PFS/DFS/MFS/RFS. The pooled analysis demonstrated that the presence of CTC-WBC clusters was significantly associated with worse OS (HR = 2.44, 95% CI: 1.74–3.40, *P *< 0.001) and PFS/DFS/MFS/RFS (HR = 1.83, 95% CI: 1.49–2.24, *P* < 0.001). Subgroup analyses based on sampling time, treatment method, detection method, detection system, cancer type, and study type consistently supported these findings. Further analyses indicated that CTC-WBC clusters were associated with larger tumor size (OR = 2.65, 95% CI: 1.58–4.44, *P* < 0.001) and higher alpha-fetoprotein levels (OR = 2.52, 95% CI: 1.50–4.22, *P* < 0.001) in hepatocellular carcinoma. However, no significant association was found between CTC-WBC clusters and TNM stage, depth of tumor invasion, or lymph node metastasis in the overall analysis.

**Conclusions:**

CTC-WBC clusters are negative predictors for OS and PFS/DFS/MFS/RFS in patients with solid tumors. Monitoring CTC-WBC levels may provide valuable information for predicting disease progression and guiding treatment decisions.

**Supplementary Information:**

The online version contains supplementary material available at 10.1186/s12885-023-11711-7.

## Introduction

Metastasis remains the leading cause of cancer-related mortality. Circulating tumor cells (CTCs), which are shed by either a primary tumor or metastasis into the blood and lymphatic systems, are precursors of metastasis in various solid cancers [1, 2]. While coping with a new and challenging microenvironment, newly disseminated cancer cells may be particularly vulnerable to immune surveillance [3]. Indeed, CTCs face a hostile environment within the bloodstream. To colonize distant organs, CTCs must overcome many obstacles, including evading immune defenses, adapting to supportive niches, infiltrating foreign tissue, surviving as latent tumor-initiating seeds, and eventually emerging to replace host tissue [3, 4]. Under particular conditions, CTCs may be found within the bloodstream in clusters with nonmalignant cells such as white blood cells (WBCs), mesenchymal stem cells (MSCs), cancer-associated fibroblasts (CAFs), and platelets [5, 6]. CTC-WBC clusters act as "hitchhikers" and are transported throughout the body by neutrophils via various mechanisms [7]. For example, neutrophils aid in tumor metastasis by deploying neutrophil extracellular traps (NETs) containing nuclear DNA, which effectively entrap CTC-WBC clusters and facilitate the progression of cancer [8]. More importantly, neutrophils play a crucial role in modulating cell cycle progression and promoting the metastatic capacity of CTCs through their systemic interaction with CTCs [9].

The prognostic value of the CTC-WBC cluster, a combination of “soil” and “seed”, has been demonstrated in multiple malignant solid tumors [10, 11]. According to the 8th edition of the American Joint Committee on Cancer (AJCC) cancer staging manual, the presence of CTC-WBCs is deemed a negative predictor for the prognosis of patients with primary or metastatic breast cancer [12]. However, some studies have indicated that preoperative CTC-WBCs do not correlate with progression-free survival (PFS) [13]. Therefore, to determine the prognostic value of CTC-WBCs and to interpret the results of available studies statistically, we performed this meta-analysis to evaluate the diagnostic accuracy and prognostic value of testing for CTC-WBC clusters in several solid cancers, including hepatocellular carcinoma (HCC) [11, 14], renal cell carcinoma (RCC) [10, 13], metastatic breast cancer (MBC) [15, 16], non-small cell lung cancer (NSCLC) [17], gastric cancer (GC) [18], colorectal cancer (CRC) [19], and small cell lung cancer (SCLC) [20].

## Methods

### Literature search strategy

On 20th December 2022, we extensively searched PubMed, Embase, and Cochrane Library for studies investigating the prognostic value of CTC-WBCs in solid-tumor patients, without time restrictions. Terms including “CTC-WBCs”, “CTC-neutrophil cluster,” and “prognosis” were jointly searched (Supplemental 1: Search strategy). To ensure comprehensive coverage and avoid overlooking valuable studies, manual searches were conducted by carefully reviewing relevant articles and references. In cases where multiple studies involved the same population, preference was given to the most recent study with complete data.

### Eligibility criteria for studies

Following the PRISMA guidelines [21], we conducted a thorough screening of the titles, abstracts, and author details of the gathered studies to identify potentially pertinent publications. We examined patients who underwent testing to detect CTC-WBC clusters before and after receiving different treatment regimens. We specifically included studies that explored the prognostic importance of CTC-WBCs in solid tumor patients and reported at least one outcome (OS and/or PFS/DFS/MFS/RFS) for detailed evaluation. To ensure the legitimacy of studies for subsequent meta-analysis, we conducted a comprehensive review of relevant articles by assessing their full texts and references using the following exclusion criteria: (1) lack of survival outcomes or insufficient data for extraction, (2) fewer than 30 cases enrolled, (3) duplicated publications, and (4) exclusion of editorials, reviews, comments, case reports, and letters. Additionally, English language was a requirement for all included studies.

### Data extraction

Two reviewers independently extracted the data. When disagreements arose, they were resolved through discussions involving the senior author (Li) to reach a consensus. Baseline characteristics recorded for each eligible study were as follows: surname of the first author, publication year, origin country, study type, cancer type, number and median/mean age of patients, median follow-up, therapies, detection platform, time points of sampling and blood volume for tests and target markers.

In addition to these characteristics, clinicopathological features were also extracted, such as TNM stage, depth of tumor invasion, lymph node metastasis, tumor size, alpha-fetoprotein (AFP) level and liver cirrhosis. The original articles included survival outcomes represented by hazard ratios (HRs) and 95% confidence intervals (CIs) for disease progression endpoints (e.g., PFS, DFS, etc.) and OS. In cases where explicit data was not provided, Engauge Digitizer v4.1 software, following the method described by Tierney et al*.*, was used to extract information from Kaplan‒Meier survival curves. All odds ratios (ORs) with corresponding 95% CIs were gathered for analysis.

### Quality assessment

The quality of the included studies was assessed independently by two authors using the Newcastle‒Ottawa Scale (NOS) for cohort studies [22], which is recommended by the Cochrane Library for observational studies. Studies scoring higher than eight were considered high quality; studies scoring less than 6 were considered low quality. The two authors reconciled disagreements by conversing and reaching a consensus.

### Statistical analysis and visualization tools

To statistically evaluate the prognostic impact of CTC-WBC clusters on cancer patient survival, we gathered individual HRs and ORs with their corresponding 95% CIs from relevant studies, with preference given to those utilizing multivariate analyses. Forest plots were used to visualize potential heterogeneity, and Cochrane's Q statistic and I^2^ statistic were computed to assess any heterogeneity observed. If it was not feasible to quantify heterogeneity (I^2^ < 50% and two-tailed *P* value > 0.1), we used fixed-effect models. If heterogeneity was present, we used random-effects models. Additionally, we conducted subgroup analyses or sensitivity analyses to investigate potential sources of heterogeneity. We evaluated publication bias using funnel plots and Egger and Begg statistics to ensure the reliability of the findings [23]. Review STATA 15.1 and RevMan 5.4 were employed for statistical analysis and visualization. The statistical significance level was set at a two-tailed *P* value threshold of < 0.05.

## Results

### Characteristics of the identified studies and quality assessment

**Fig. 1:**
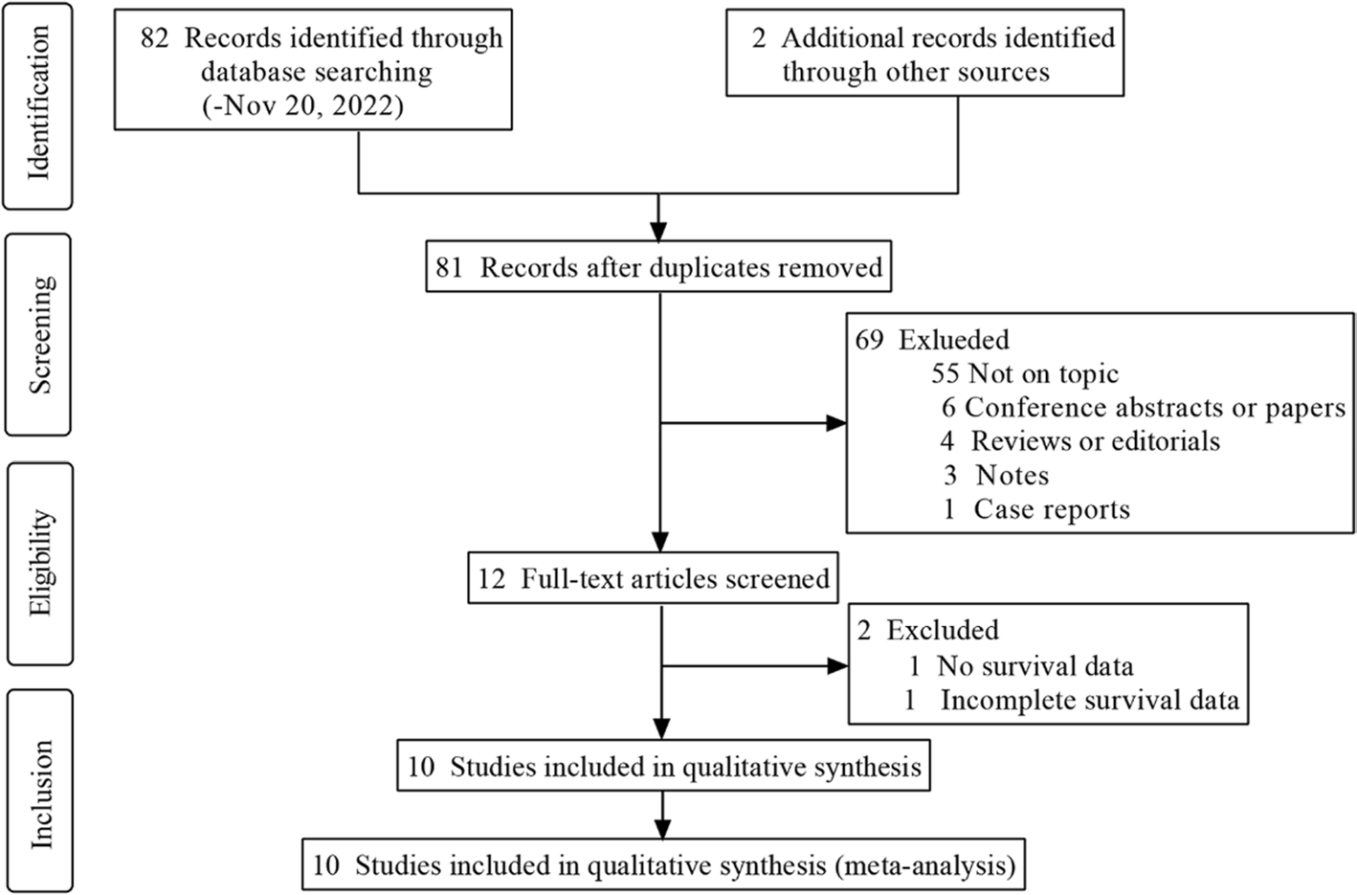
Flow diagram of the literature screening

The process of literature screening is presented in Fig. 1, and 84 studies were initially retrieved. After removing 3 duplicates and excluding 71 studies for various reasons, 12 full-text articles were read in detail. Ten studies including 1471 patients were ultimately included for further analysis. Of the 1471 patients, 1111 (75.5%, from six studies) were screened for CTC-WBC clusters using a blood volume of 5 mL, 94 (6.4%, from two studies) were screened using 6 mL, and 266 (18.1%, from two studies) were screened using 7.5 mL. In two studies [10, 18], blood samples were collected from patients after local or systemic therapy. Five other studies [11, 14, 15, 17, 19] focused on pretreatment samples. Three studies [13, 16, 20] collected blood samples from patients at multiple time points, allowing for confirmation of the prognostic value of positive results at different stages (Table 1). The techniques used for CTC-WBC detection included CanPatrol, CellSearch, and Cytelligen systems. The main characteristics of the identified studies are summarized in Table 1.

**Table 1:** Characteristics of the identified studies

Study	Country	Study type	Cancer type	Patients(n)	Median age(years)	Median follow-up	Therapies	Endpoints	Quality
Chen 2022 [14]	China	retrospective	HCC	136	50(25–78)	18.0(2–72)	local therapy (surgical resection, interventional or radiofrequency ablation therapy)	RFS	7
Guan(1) 2021 [10]	China	retrospective	RCC	163	NR	NR	local therapy (surgery)	MFS/OS	8
Guan(2) 2021 [15]	China	prospective	MBC	135	51(27–73)	36.0(27.8–44.3)	systemic therapy (chemotherapy)	PFS	7
Jansson 2016 [16]	Sweden	prospective	MBC	52	60(40–83)	12(5–44), 10(1–42), 15(1–38)	systemic therapy (endocrine/chemotherapy)	PFS/OS	7
Li 2022 [17]	China	retrospective	NSCLC	61	NR	8.5(2.1–14.7)	local therapy (surgery)/systemic therapy	PFS	6
Luo 2020 [11]	China	retrospective	HCC	214	53	52(18–78)	local therapy (surgery)	DFS/OS	7
Qiu 2022 [18]	China	retrospective	GC	217	59(29–89)	18.5(4–30)	local therapy (surgery)	OS	8
Wang 2021 [13]	China	retrospective	RCC	131	NR	24(6–61)	local therapy (surgery)	PFS	6
Xu 2022 [19]	China	retrospective	CRC	329	58(16–88)	30(11–43)	local therapy (surgery)	PFS/OS	7
Zhu 2021 [20]	China	prospective	SCLC	33	63(43–69)	20(2.8–30.2)	systemic therapy (chemotherapy)	PFS/OS	7
Study	Detection method	Detection system	Marker	Sampling time	Positive rate	Blood(mL)	Cutoff		
Chen 2022 [14]	RNA-ISH	CanPatrol	epithelial markers: EpCAM/CKs mesenchymal markers: Vimentin/Twist leukocyte marker: CD45	baseline	31.6%	5	1		
Guan(1) 2021 [10]	RNA-ISH	CanPatrol	epithelial markers: EpCAM, CK8, CK18, and CK19 mesenchymal markers: Vimentin/Twist leukocyte marker: CD45	3 months after surgery	11.7%	5	0		
Guan(2) 2021 [15]	RNA-ISH	CanPatrol	epithelial markers: EpCAM and CK8, CK18, and CK19 mesenchymal markers: Vimentin/Twist leukocyte marker: CD45	baseline	5.9%,	5	0		
Jansson 2016 [16]	ICC	CellSearch	epithelial cells: CK8, CK18, and CK19 leukocyte marker: CD45	baseline,1–3, and 6 months after chemotherapy or until disease progression	67.3% 35.3% 29.6%	7.5	0		
Li 2022 [17]	SE-iFISH	Cytelligen	CTC: Vimentin leukocyte marker: CD45	baseline	29.3%	6	0, 3		
Luo 2020 [11]	RNA-ISH	CanPatrol	epithelial cells: EpCAM and CK8, CK18, and CK19 mesenchymal cells: Vimentin/Twist leukocyte marker: CD45	baseline	41.6%	7.5	2		
Qiu 2022 [18]	RNA-ISH	CanPatrol	epithelial biomarkers: EpCAM and CK8, CK18, and CK19 mesenchymal biomarkers: Vimentin/Twist leukocyte marker: CD45	after radical resection	13.4%	5	0		
Wang 2021 [13]	RNA-ISH	CanPatrol	epithelial markers: EpCAM, CK8, CK18, and CK19 mesenchymal markers: Vimentin/Twist leukocyte marker: CD45	baseline	19.1	5	0		
Xu 2022 [19]	RNA-ISH	CanPatrol	epithelial markers: EpCAM, CK8, CK18, and CK19 mesenchymal markers: Vimentin/Twist leukocyte marker: CD45	baseline and 3 months after surgery	11.5% 13.0%	5	0		
Zhu 2021 [20]	SE-iFISH	Cytelligen	cancer stem cell (CSC) marker: CD44 epithelial-to-mesenchymal transition (EMT) marker: vimentin leukocyte marker: CD45	baseline, and following two cycles of chemotherapy	24.1% 48.1%	6	0		

*CTC-WBC* Circulating tumor cell-white blood cell, *DFS* Disease-free survival, *GC* Gastric cancer, *HCC* Hepatocellular carcinoma, *ICC* Immunocytochemistry, *MBC* Metastatic breast cancer, *MFS* Metastasis-free survival, *NR* Not reported, *NSCLC* Non-small cell lung cancer, *OS* Overall survival, *PFS* Progression-free survival, *RCC* Renal cell carcinoma, *RNA-ISH* RNA in situ gybridization, *SCLC* Small cell lung cancer, *SE-iFISH* Serial expression in situ hybridization

### Clinicopathological features

**Fig. 2:**
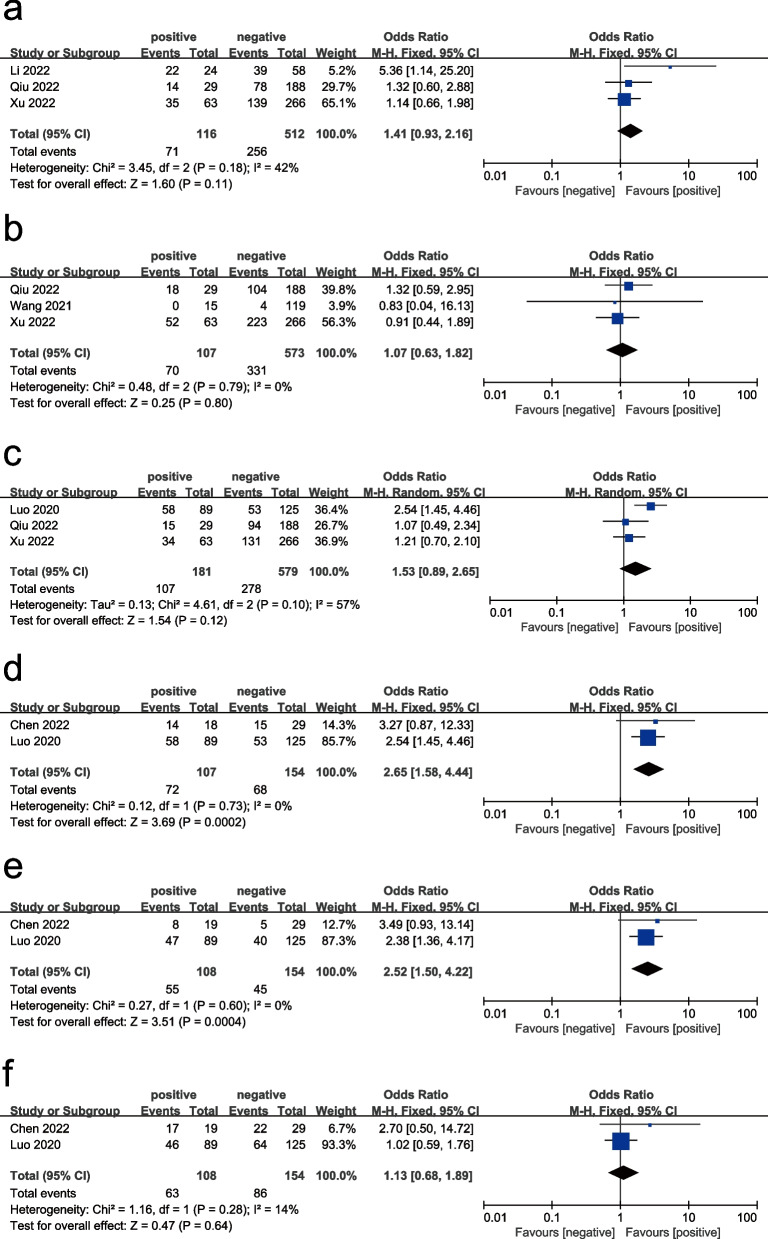
Forest plots showing the odds ratios for TNM stage (**a**), depth of tumor invasion (**b**), lymph node metastases (**c**), tumor size (**d**), AFP level (**e**), and liver cirrhosis (**f**)

Clinicopathological features, including TNM stage, depth of tumor invasion, lymph node metastases, tumor size, AFP level, and liver cirrhosis, were analyzed for associations with CTC-WBCs. The results are summarized in Table 2, revealing that CTC-WBCs were not significantly associated with TNM stage, depth of tumor invasion, or lymph node metastases (OR = 1.20, 95% CI: 0.76–1.88, *P* = 0.44; OR = 1.07, 95% CI: 0.63–1.82, *P* = 0.80, and OR = 1.53, 95% CI: 0.89–2.65, *P* = 0.12, respectively). Only two studies were used to assess the relationship between CTC-WBCs and tumor size, AFP level, and liver cirrhosis in liver cancer. CTC-WBCs were significantly associated with larger tumors and high AFP levels (OR = 2.65, 95% CI: 1.58–4.44, *P* < 0.001; OR = 2.52, 95% CI: 1.50–4.22, *P* < 0.001, respectively) but not significantly associated with liver cirrhosis (OR = 1.13, 95% CI: 0.68–1.89, *P* = 0.64) (Fig. 2).)

**Table 2:** Associations of CTC-WBCs with clinicopathological features

Outcomes	Study	Odd ratio	Z and P for hazard ratio	Heterogeneity (I^2^, P)	Publication bias
TNM stage (III-IV vs. Stage I-II)	Li, Qiu, Xu	1.20 (0.76–1.88)	Z = 0.78, *P* = 0.44	0%, 0.77	/
Depth of tumor invasion (T3-T4 vs. T1-T2)	Qiu, Wang, Xu	1.07 (0.63–1.82)	Z = 0.25, *P* = 0.80	0%, 0.79	/
Lymph node metastases (Yes vs. no)	Luo, Qiu, Xu	1.53(0.89–2.65)	Z = 1.54, *P* = 0.12	57%,0.10	/
Tumor size(> 5 vs. ≤ 5)	Chen, Luo	2.65(1.58–4.44)	Z = 3.69, *P* < 0.001	0%, 0.73	Begg’s Test = 1.000 Egger’s test = /
AFP level (≥ 400 vs. < 400)	Chen, Luo	2.52(1.50–4.22)	Z = 3.51, *P* < 0.001	0%, 0.60	Begg’s Test = 1.000 Egger’s test = /
Liver cirrhosis (Yes vs. no)	Chen, Luo	1.13 (0.68–1.89)	Z = 0.47, *P* = 0.64	14%, 0.28	/

*Abbreviations*: *I*^*2*^ degree of heterogeneity

### Quality assessment

The methodological quality of the included studies was evaluated independently by two reviewers based on NOS criteria. All of the studies were considered to be of medium or high quality, as indicated by scores of at least six (Table 1).

### Impact of CTC-WBC clusters on OS and PFS/DFS/MFS/RFS

**Fig. 3:**
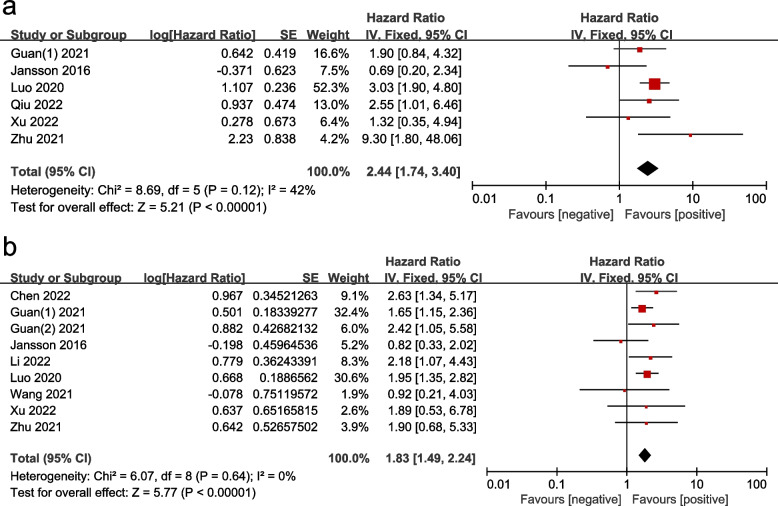
Forest plots showing the hazard ratios for OS and PFS/DFS/MFS/RFS

HRs for OS were extracted from six studies, with values ranging from 0.69 to 9.30. As there was no heterogeneity among the studies (*P* = 0.12, I^2^ = 42%), a fixed model was used to calculate the pooled HR. The results shown in Fig. 3a indicate that the presence of CTC-WBCs was significantly associated with OS (HR = 2.44, 95% CI: 1.74–3.40, *P* < 0.001), demonstrating that the risk of death increased dramatically in the CTC-WBC-positive group. Additionally, nine studies reported HRs for disease progression endpoints (e.g., PFS, DFS, etc.), with values ranging from 0.82 to 2.65. The pooled HR in Fig. 3b shows that the presence of CTC-WBCs was significantly associated with PFS/DFS/MFS/RFS (HR = 1.83, 95% CI: 1.49–2.24, *P* < 0.001), with the CTC-WBC-positive group having a significantly higher risk of disease progression. Sensitivity analyses revealed that, with the exception of the study by Jansson et al*.* [16], no other study substantially dominated the results (Supplementary Fig. 1). Given the potential impact of this study, we made a decision to exclude it from the final analysis based on methodological issues. The comparison of results before and after the exclusion demonstrates the robustness of our findings, and the final conclusions are less susceptible to the influence of the excluded study (HR = 2.69, 95% CI: 1.90–3.81, *P* < 0.001).

### Subgroup analyses

#### Sampling time

Blood samples were collected from cancer patients before and after treatment. Studies with extractable data for OS were separated into the “pretherapy” subgroup and the “posttherapy” subgroup to investigate the effect of sampling time on the prognostic value of CTC-WBCs. Based on the results, CTC-WBCs obtained before treatment were significantly associated with disease progression endpoints (e.g., PFS, DFS, etc.) (HR = 1.91, 95% CI: 1.51–2.42, *P *< 0.001, Table 3), and CTC-WBCs obtained after treatment were significantly associated with OS (HR = 2.62, 95% CI: 1.51–4.56, *P* = 0.001, Table 3). According to the results of sensitivity analyses, we decided to exclude one study and reanalyze the data (Supplementary Fig. 2). The final results showed CTC-WBCs to be significantly associated with OS and PFS/DFS/MFS/RFS, regardless of sampling time (Table 3).

**Table 3:** Subgroup analyses of HRs for OS and PFS/MFS/RFS/DFS

	Studies	Hazard ratio	Z and P for hazard ratio	Heterogeneity (I^2^, P)	Publication bias
**OS**					
Sampling time					
Pretherapy	Jansson, Luo, Xu, Zhu	2.16(0.88–5.3)	Z = 1.69, *P* = 0.092	64.0%, 0.040	Begg’s Test = 0.734; Egger’s test = 0.261
Pretherapy^a^	Luo, Xu, Zhu	2.98(1.96–4.53)	Z = 2.76, *P* = 0.006	41.4%, 0.182	Begg’s Test = 1.000; Egger’s test = 0.416
Posttherapy	Guan(1), Jansson, Qiu, Zhu	2.62 (1.51–4.56)	Z = 3.34, *P* = 0.001	3.6%, 0.375	Begg’s Test = 0.089; Egger’s test = 0.295
Posttherapy^a^	Guan(1), Qiu, Zhu	3.43(1.38–4.26)	Z = 3.09, *P* = 0.002	0%, 0.592	Begg’s Test = 0.296; Egger’s test = 0.382
Treatment method					
Local therapy	Guan(1), Qiu, Xu	1.97(1.13–3.43)	Z = 2.40, *P* = 0.016	0%, 0.710	Begg’s Test = 1.000; Egger’s test = 0.386
Systematic therapy	Jansson, Luo, Zhu	3.42(2.21–5.32)	Z = 3.07, *P* = 0.002	38.1%, 0.199	Begg’s Test = 0.296; Egger’s test = 0.054
Detection method					
RNA-ISH	Guan(1), Luo, Qiu, Xu	2.55(1.78–3.63)	Z = 5.15, *P* < 0.001	0%, 0.578	Begg’s Test = 0.308; Egger’s test = 0.021
Detection system					
CanPatrol	Guan(1), Luo, Qiu,Xu	2.55(1.78–3.63)	Z = 5.15, *P* < 0.001	0%, 0.578	Begg’s Test = 0.308; Egger’s test = 0.021
Study type					
Prospective	Jansson, Zhu	11.44 (2.71–48.37)	Z = 3.31, *P* = 0.001	0%, 0.605	Begg’s Test = 1.000; Egger’s test = /
Retrospective	Guan(1), Luo, Qiu, Xu	2.55 (1.78–3.63)	Z = 5.15, *P* < 0.001	0%, 0.578	Begg’s Test = 0.308; Egger’s test = 0.021
**PFS/DFS/MFS/RFS**					
Sampling time					
Pretherapy	Chen, Guan(2), Jansson, Li, Luo, Wang, Xu, Zhu	1.91 (1.51–2.42)	Z = 5.42, *P* < 0.001	0%, 0.584	Begg’s Test = 0.386; Egger’s test = 0.019
Pretherapy^a^	Chen, Guan(2), Li, Luo, Wang, Xu, Zhu	2.04(1.60–2.60)	Z = 5.73, *P* < 0.001	0%, 0.922	Begg’s Test = 0.881; Egger’s test = 0.010
Posttherapy	Guan(1), Jansson, Wang, Zhu	1.64 (0.89–3.00)	Z = 1.44, *P* = 0.151	56.4%, 0.0.076	Begg’s Test = 0.734; Egger’s test = 0.301
Posttherapy^a^	Guan(1), Wang, Zhu	1.78 (1.29–2.44)	Z = 2.49, *P* = 0.013	35.2%, 0.214	Begg’s Test = 0.296; Egger’s test = 0.691
Treatment method					
Local therapy	Chen, Guan(1),Luo, Wang, Xu	1.95(1.57–2.43)	Z = 5.99, *P* < 0.001	0%, 0.447	Begg’s Test = 0.086; Egger’s test = 0.430
Systematic therapy	Guan(2), Jansson, Li, Zhu	1.99(1.24–3.19)	Z = 1.99, *P* = 0.047	33.0%, 0.214	Begg’s Test = 0.308; Egger’s test = 0.024
Detection method					
RNA-ISH	Chen, Guan(1), Guan(2), Luo, Qiu, Wang, Xu	1.88(1.50–2.36)	Z = 5.51, *P* = 0.047	0%, 0.742	Begg’s Test = 0.707; Egger’s test = 0.935
SE-iFISH	Li, Zhu	2.09 (1.16–3.74)	Z = 2.46, *P* = 0.014	0%, 0.465	Begg’s Test = 1.000; Egger’s test = /
Detection system					
CanPatrol	Chen, Guan(1), Guan(2), Luo, Xu	1.91 (1.54–2.38)	Z = 5.86, *P* < 0.001	0%, 0.770	Begg’s Test = 0.221; Egger’s test = 0.172
Cytelligen	Li, Zhu	1.86 (1.05–3.29)	Z = 2.14, *P* = 0.033	0%, 0.465	Begg’s Test = 1.000; Egger’s test = /
Cancer type					
HCC	Chen, Luo	2.09 (1.51–2.89)	Z = 4.45, *P* < 0.001	0%, 0.448	Begg’s Test = 1.000; Egger’s test = /
MBC	Guan(2), Jansson	0.76 (0.05–11.74)	Z = 0.20, *P* = 0.841	0%, 0.460	Begg’s Test = 1.000; Egger’s test = /
RCC	Guan(1),Wang	1.60 (1.31–2.26)	Z = 2.63, *P* = 0.009	0%, 0.454	Begg’s Test = 1.000; Egger’s test = /
Study type					
Prospective	Guan(2), Jansson, Zhu	1.31 (0.46–3.75)	Z = 0.51, *P* = 0.609	56.0%, 0.103	Begg’s Test = 0.297; Egger’s test = 0.138
Retrospective	Chen, Guan(1), Li, Luo, Wang, Xu	1.88 (1.52–2.32)	Z = 5.93, *P* < 0.001	0%, 0.769	Begg’s Test = 1.000; Egger’s test = 0.046

*Abbreviations*: *HCC* Hepatocellular carcinoma, *I2* Degree of heterogeneity, *MBC* Metastatic breast cancer, *RCC* Renal cell carcinoma, *RNA-ISH* RNA in situ hybridization, *SE-iFISH* Serial expression in situ hybridization

^a^subgroup analyses reflecting the results obtained after excluding the studies that had a significant impact on the overall findings

#### Treatment method

The ten studies were categorized into two groups based on the different treatment methods used: a "local therapy" group (including patients who underwent surgery, intervention, or ablation but excluding those who received chemotherapy or any other systemic therapy) and a "systematic therapy" group (including patients who received chemotherapy, targeted therapy, endocrine therapy, or immunotherapy). Among patients who underwent local therapy, CTC-WBCs were significantly associated with OS (HR = 1.97, 95% CI: 1.13–3.43, *P* = 0.016, Table 3) as well as PFS, DFS, MFS and RFS (HR = 1.95, 95% CI: 1.57–2.43, *P* < 0.001, Table 3). In patients who received systematic therapy, the HR and 95% CI for OS were 3.42 and 2.21–5.32 (*P* = 0.002), and those for disease progression endpoints (e.g., PFS, DFS, etc.) were 1.99 and 1.24–3.19 (*P* = 0.047, Table 3), respectively.

#### Detection method

In the subgroup analysis stratified by detection method, the prognostic value of CTC-WBCs for both OS and PFS/DFS/MFS/RFS were found to be significant in the RNA in situ hybridization(RNA-ISH) subgroup, with HR of 2.55 (95%CI: 1.78–3.63, *P* < 0.001, Table 3) for OS and 1.88 (95% CI: 1.50–2.36, *P* = 0.047, Table 3) for disease progression endpoints (e.g., PFS, DFS, etc.). Additionally, in the specific enrichment-immunofluorescence in situ hybridization (SE-iFISH) subgroup, the results indicated a significant association between CTC-WBCs and disease progression endpoints, with an HR of 2.09 (95% CI: 1.16–3.74, *P* = 0.014, Table 3).

#### Detection system

We compared various systems for detecting CTC-WBCs, including the CanPatrol and Cytelligen systems. In the CanPatrol subgroup, we observed a significant association between CTC-WBC detection and OS (HR = 2.55, 95% CI: 1.78–3.63, *P* < 0.001, Table 3), as well as PFS, DFS, MFS and RFS (HR = 1.91, 95% CI: 1.54–2.38, *P* < 0.001, Table 3). We also found a significant association with PFS, DFS, MFS and RFS in the Cytelligen subgroup (HR 1.86, 95% CI: 1.07–3.29, *P* = 0.033, Table 3).

#### Cancer type

The included studies were categorized into three groups according to cancer type: an HCC group, an MBC group, and an RCC group. For HCC and RCC patients, CTC-WBCs were significantly associated with PFS/DFS/MFS/RFS (HR = 2.09, 95% CI: 1.51–2.89, *P* < 0.001; HR = 1.60, 95% CI: 1.31–2.26, *P* = 0.009, respectively). However, for MBC patients, CTC-WBCs were not significantly associated with survival (HR = 0.76, 95% CI: 0.05–11.74, *P* = 0.841, Table 3). The prognostic impact of CTC-WBC clusters was also explored in individual cancer types, each represented by a singular study, which provided valuable insights despite the limited number of reports. In NSCLC, the study [17]revealed a significant correlation of CTC-WBCs with PFS (HR = 2.18, 95% CI: 1.07–4.43, *P* = 0.031). Qiu et al. [18] identified a noteworthy association of CTC-WBCs with OS in GC (HR = 2.553, 95% CI: 1.008–6.465, *P* = 0.048). Xu et al. [19] observed a similar trend for PFS in CRC (HR = 1.89, 95% CI: 1.02–3.51, *P* = 0.042), though the OS association was not significant. Additionally, Zhu et al. [20] found that in SCLC, CTC-WBC clusters were a significant prognostic factor for OS both before treatment (HR = 9.3, 95% CI: 1.4–48, *P* = 0.0079) and after two chemotherapy cycles (HR = 4.4, 95% CI: 1.1–18, *P* = 0.041), highlighting their consistent prognostic value regardless of treatment stage.

#### Study type

In our stratified analysis by study design, both prospective and retrospective studies were examined to evaluate their impact on the prognostic significance of CTC-WBC clusters.

Prospective studies, as represented by Jansson et al. [16] and Zhu et al. [20], showed a significant association of CTC-WBCs with OS (HR = 11.44, 95% CI: 2.71–48.37, *P* = 0.001, Table 3). The retrospective studies [10, 11, 18, 19] also demonstrated a notable association with OS (HR = 2.55, 95% CI: 1.78–3.63, *P* < 0.001, Table 3). For disease progression endpoints, the prospective group reported an HR of 1.31 (95% CI: 0.46–3.75, *P* = 0.609). In contrast, the retrospective group indicated a significant relationship with these outcomes (HR = 1.88, 95% CI: 1.52–2.32, *P* < 0.001, Table 3).

### Publication bias

**Fig. 4:**
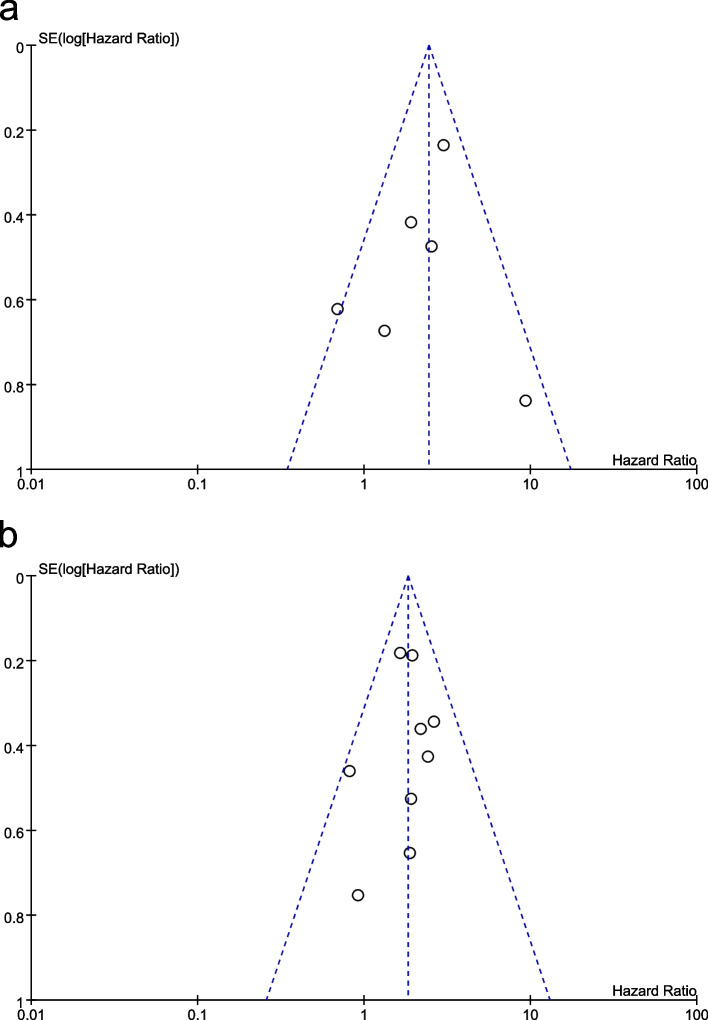
Funnel plot illustrating meta-analysis of OS (**a**) and PFS/DFS /MFS/RFS (**b**). SE: standard error

A funnel chart (Fig. 4) and the results of Begg's and Egger's test analysis (Table 3) suggested no significant publication bias.

## Discussion

To our knowledge, this study constitutes the first pooled analysis evaluating the prognostic significance of CTC-WBCs. Our systematic review and meta-analysis included three prospective [15, 16, 20] and seven retrospective studies [10, 11, 13, 14, 17–19], involving a total of 1471 patients with seven different types of solid cancer. The main findings of the current study indicate that the presence of CTC-WBCs is significantly associated with worse OS and PFS/DFS/MFS/RFS in patients with solid tumors. The HRs were 2.44 (95% CI: 1.74–3.40, *P* < 0.001) for OS and 1.83 (95% CI: 1.49–2.24, *P* < 0.001) for disease progression endpoints. Similar results were obtained in subgroup analyses based on sampling time, treatment method, detection method, detection system, cancer type and study type. Moreover, sensitivity analyses confirmed the reliability of the findings, and no significant indication of publication bias was found. These results suggest that the presence of CTC-WBCs could indicate tumor spread and predict a more advanced tumor stage. Monitoring CTC-WBC levels before and after treatment may provide valuable information for predicting disease progression and determining appropriate treatment strategies.

Considering the potential impact of sampling time on study outcomes, we conducted a stratified analysis based on sampling time. The results of the included studies showed considerable heterogeneity when stratifying by sampling time. Our sensitivity analysis showed that Jansson's research had the most significant impact on the overall results. It was found that this study utilized the CellSearch detection system and employed immunocytochemistry (ICC) for CTC-WBC detection. Interestingly, this was the sole study that utilized ICC, potentially contributing to the observed heterogeneity in the results. In addition, it is important to consider that CellSearch may underestimate CTCs that have undergone epithelial-to-mesenchymal transition (EMT). Therefore, we excluded Jansson's study and found that the results for OS and PFS/DFS/MFS/RFS were statistically significant both before and after treatment. Our analysis showed that the presence of CTC-WBCs before treatment indicates tumor spread and can predict a later tumor stage. On the other hand, the CTC-WBCs remaining after clinical intervention can act as a "seed" of a tumor, leading to earlier recurrence and worse prognosis. These findings suggest that monitoring levels of CTC-WBCs before and after treatment can provide valuable information for predicting disease progression and prognosis.

Regarding treatment options, our meta-analysis demonstrated that CTC-WBC positivity can serve as an excellent prognostic indicator for both local and systemic therapies. Chemotherapy or targeted therapy can impact the phenotype of tumor cells by targeting those cells with proliferative activity, causing their death. However, cancer cells with weak proliferative activity may survive and develop resistance to systematic therapy, which can lead to tumor recurrence through immunoediting [24, 25]. According to Davies et al. [26], liquid biopsy of CTCs can provide real-time information about the heterogeneous nature of a tumor, making it a potentially ideal biomarker for predicting response to docetaxel. In addition, some studies have suggested that surgery can temporarily disseminate CTC-WBCs, resulting in subsequent spread of CTCs and worse survival outcomes [27–29]. Thus, our findings indicate that CTC-WBCs can provide valuable information not only for predicting prognosis but also for determining appropriate treatment strategies.

Accurate and reliable detection methods and systems are essential for identifying CTC-WBCs, minimizing misdiagnosis and missed detection. Each method offers unique technical advantages, providing valuable molecular or morphological information. By selecting comparable methods and systems, researchers ensure consistent and comparable results, enabling effective comparison and integration of findings across studies. In subgroup analysis, we found consistent prognostic value of CTC-WBCs across analyses stratified by detection method and system. Based on available literature, RNA-ISH and SE-iFISH offer the advantage of high sensitivity and specificity, allowing for both molecular characterization and enumeration of CTC-WBCs [30–32]. In the realm of detection systems, both CanPatrol and Cytelligen offer cutting-edge tools for CTC-WBC analysis. CanPatrol excels in molecular characterization through RNA-ISH, while Cytelligen specializes in immunomagnetic enrichment and high-quality ICC for detailed morphological information. Researchers can choose between these high-end systems based on their specific goals, whether it's a focus on molecular insights or intricate morphological analysis of CTC-WBCs [33, 34].

To investigate the prognostic value of CTC-WBCs across different types of cancer, subgroup analyses were performed for various cancer types. According to the results, CTC-WBC clusters may not have significant prognostic value for patients with MBC, possibly due to the limited sample size and study design. However, the heterogeneity observed in subgroup analyses between two studies might also be attributed to differences in patient sampling time and treatment plans.

We also conducted stratified analyses based on study design, categorizing studies as either prospective or retrospective. Both prospective and retrospective studies consistently demonstrated a significant association between CTC-WBC clusters and OS. This consistency reaffirms the robustness of CTC-WBC clusters as a reliable prognostic marker, unaffected by inherent research biases. However, when examining PFS, DFS, MFS, and RFS within prospective studies, a notable impact of CTC-WBC clusters on these outcomes was not observed. This divergence may be attributed to differences in prospective study design, such as the variability in disease progression rates, the timing and sensitivity of outcome measurements, or the influence of intervening clinical factors that are more dynamically controlled in a prospective setting.

In terms of clinicopathological features, no significant association was observed with TNM stage, depth of tumor invasion or lymph node metastases. In general, patients at late stages and with lymph node metastases have poorer prognoses [35]. A possible reason for the lack of association is the limited number of cases, and more studies are required to explain the inconsistent result. For HCC, CTC-WBCs in blood were significantly associated with larger tumor size and higher AFP levels. The larger the size of a tumor is, the faster it progresses or the longer it grows. The level of AFP also to a certain extent reflects tumor size, and its dynamic change has a particular relationship with cancer. AFP is reportedly a sensitive indicator for treatment effect and prognosis [36].

Understanding the characteristics of the interaction between cancer cells and immune cells is essential for developing new cancer treatment methods. The number of CTC-WBCs is typically low, usually in single cells or clusters [18]. As mentioned above, some studies have found that CTC-WBCs can exist in association with leukocytes (a large class of immune cells, including neutrophils, eosinophils, basophils, monocytes and lymphocytes) [37]. As the predominant white blood cell population in the bloodstream of humans, neutrophils are a critical component of the innate immune system. Tumor-associated neutrophils (TANs) play a significant role in the growth and metastasis of cancer cells within the tumor microenvironment. They exert their influence through both direct and indirect mechanisms. Directly, TANs interact with cancer cells, while indirectly, they modify the tumor microenvironment to support cancer cell proliferation and dissemination [38]. These TANs exhibit a gene expression pattern similar to that of pretumor cells, expressing genes that facilitate angiogenesis, remodel the surrounding tissue, promote cancer cell metastasis, and suppress the immune system's response, ultimately promoting tumor growth [37, 39]. In 2019, Szczerba et al*.* [7]conducted a groundbreaking study focused on isolating CTC-WBCs and corresponding cancer cells from breast cancer patients and mouse models. They performed meticulous transcriptome analyses, comparing the profiles of CTC-WBCs associated with neutrophils to those of CTC-WBCs alone. This investigation revealed differentially expressed genes that play crucial roles in cell cycle progression and efficient metastasis facilitation. Importantly, the presence of CTC-WBC clusters was found to be significantly associated with poor patient outcomes, highlighting the urgent need for effective strategies in treating tumor metastasis. However, despite these significant findings, many aspects regarding the features, functions, and molecular characteristics of WBCs related to CTCs remain unclear. The precise nature of CTC-WBC clusters and the underlying principles governing the interplay between CTCs and WBCs during hematogenous spread remain largely unexplored. Therefore, further extensive research efforts are necessary to unravel the intricate mechanisms involved and to enhance diagnostic accuracy in this field.

Our analysis reveals a significant link between CTC-WBCs and unfavorable survival outcomes. However, it's important to acknowledge the limitations of our meta-analysis. One limitation is the variation in CTC-WBC sampling standard and detection systems across the included studies, which may impact positive rates and survival analyses. The absence of a standardized definition for a positive sample also poses challenges in accurately interpreting findings. Furthermore, the consolidation of survival outcomes like PFS, DFS, MFS and RFS in our meta-analysis was necessitated by limited data for each endpoint. Despite their clinical differences, aggregating these metrics was crucial for maintaining statistical robustness and providing a holistic view of disease progression. Consequently, a comprehensive large-scale prospective clinical trial with an extended follow-up period is crucial. More research is needed to understand the mechanisms, establish standardized cutoff points for detecting CTC-WBCs, and explore tumor-immune cell interactions. This includes studying the molecular characteristics, functional roles, and therapeutic potential of CTC-WBC clusters, as well as how monitoring them can guide treatment decisions.

## Conclusion

In conclusion, this comprehensive meta-analysis underscores the prognostic significance of circulating tumor cell associated white blood cell CTC-WBC clusters in various solid cancers. The presence of CTC-WBC clusters is consistently associated with unfavorable prognosis, highlighting their potential as valuable biomarkers for predicting cancer prognosis and guiding treatment decisions. The findings emphasize the intricate interplay between tumor cells and the immune microenvironment during metastasis. Despite the heterogeneity in detection methods and cancer types, the consistent trends observed in subgroup analyses suggest the clinical relevance of CTC-WBC clusters across different contexts. For CTC-WBC clusters to be widely accepted as prognostic tools, it is critical to standardize how we detect them and define meaningful cutoff points. Exploring how these clusters form and their role in cancer will further this goal. Conducting large, long-term studies will confirm their relevance in clinical settings, ultimately improving how we predict and treat advanced cancer for better patient outcomes.

### Supplementary Information

**Additional file 1:** **Supplementary Table 1. **Search strategy. **Supplementary Figure 1. **Sensitivity analysis of OS (a) and PFS/DFS/RFS/MFS (b). **Supplementary Figure 2. **Sensitivity analysis of OS for the pretherapy subgroup (a) based on sampling time and PFS/DFS/RFS/MFS for the posttherapy subgroup (b).

## Data Availability

The data that support the findings of this study are available from the corresponding author upon reasonable request.

## Abbreviations

AFP: Alpha-fetoprotein
AJCC: American joint committee on cancer
CAFs: Cancer-associated fibroblasts
CIs: Confidence intervals
CRC: Colorectal cancer
CTCs: Circulating tumor cells
DFS: Disease-free survival
EMT: Epithelial-to-mesenchymal transition
EpCAM: The epithelial cell adhesion molecule
GC: Gastric cancer
HCC: Hepatocellular carcinoma
HRs: Hazard ratios
ICC: Immunocytochemistry
MBC: Metastatic breast cancer
MFS: Metastasis-free survival
MSCs: Mesenchymal stem cells
NETs: Neutrophil extracellular traps
NOS: Newcastle‒Ottawa Scale
NSCLC: Non-small cell lung cancer
ORs: Odds ratios
OS: Overall survival
PFS: Progression-free survival
RCC: Renal cell carcinoma
RFS: Recurrence-free survival
RNA-ISH: RNA in situ hybridization
SCLC: Small cell lung cancer
SE-iFISH: Specific enrichment-immunofluorescence in situ hybridization
TANs: Tumor-associated neutrophils
WBCs: White blood cells
