# Supplementary material for: Prognostic value of circulating tumor cells associated with white blood cells in solid cancer: a systematic review and meta-analysis of 1471 patients with solid tumors
Source: BMC Cancer. 2023 Dec 12;23:1224. doi: 10.1186/s12885-023-11711-7 (PMC10717563; doi:10.1186/s12885-023-11711-7)

**Supplementary Online Content**

**Supplementary Table 1.** Search strategy

**Supplementary Figure 1.** Sensitivity analysis of OS (a) and PFS/DFS/RFS/MFS (b)

**Supplementary Figure 2.** Sensitivity analysis of OS for the pretherapy subgroup (a) based on sampling time and PFS/DFS/RFS/MFS for the posttherapy subgroup (b)

This supplementary material has been provided by the authors to give readers additional information about their work.

**Supplementary Table 1.** Search strategy

| **Database** | Search Strategy |
| --- | --- |
| **Pubmed (to December 20, 2022)** | (CTC-WBC*[Title/Abstract] OR CTC White Blood Cell*[Title/Abstract] OR CTC-associated WBC*[Title/Abstract] OR CTC-WBC cluster*[Title/Abstract] OR circulating tumor cell-associated white blood cell cluster*[Title/Abstract] OR CTC-white blood cell cluster*[Title/Abstract] OR CTC-neutrophil cluster*[Title/Abstract]) AND ("Prognosis"[Mesh] OR prognostic[Title/Abstract] OR survival[Title/Abstract] OR predict[Title/Abstract] OR recurrence[Title/Abstract] OR mortality[Title/Abstract] OR metastasis[Title/Abstract]) |
| **Embase (to December 20, 2022)** | ('CTC-WBC*':ab, ti OR 'CTC White Blood Cell*':ab, ti OR 'CTC-associated WBC*':ab, ti OR 'CTC-WBC cluster*':ab, ti OR 'circulating tumor cell-associated white blood cell cluster*':ab, ti OR 'CTC-white blood cell cluster*':ab, ti OR 'CTC-neutrophil cluster*':ab, ti) AND ('prognosis'/exp OR 'prognostic':ab, ti or 'survival':ab, ti or 'predict':ab, ti or 'recurrence':ab, ti or 'mortality':ab, ti or ' metastasis':ab, ti) |
| **Cochrane Libray (to December 20, 2022)** | 1、(CTC-WBC):ti, ab,kw OR (CTC-WBCs):ti, ab,kw OR (CTC White Blood Cell):ti, ab,kw OR (CTC White Blood Cells):ti, ab,kw OR (CTC-associated WBC):ti, ab,kw OR (CTC-associated WBCs):ti, ab,kw  OR (CTC-WBC cluster):ti, ab,kw OR (CTC-WBC clusters):ti, ab,kw OR (circulating tumor cell-associated white blood cell cluster):ti, ab,kw OR (circulating tumor cell-associated white blood cell clusters):ti, ab,kw OR (CTC-white blood cell cluster):ti, ab,kw OR (CTC-white blood cell clusters):ti, ab,kw OR (CTC-neutrophil cluster):ti, ab,kw OR (CTC-neutrophil clusters):ti, ab,kw  2、MeSH descriptor: [Prognosis] explode all trees  3、(prognostic):ti, ab,kw OR (survival):ti, ab,kw OR (predict):ti, ab,kw OR (recurrence):ti, ab,kw OR (mortality):ti, ab,kw OR (metastasis):ti, ab,kw  4、#1 AND (#2 OR #3) |

**Supplementary Figure 1.** Sensitivity analysis of OS (a) and PFS/DFS/RFS/MFS (b)


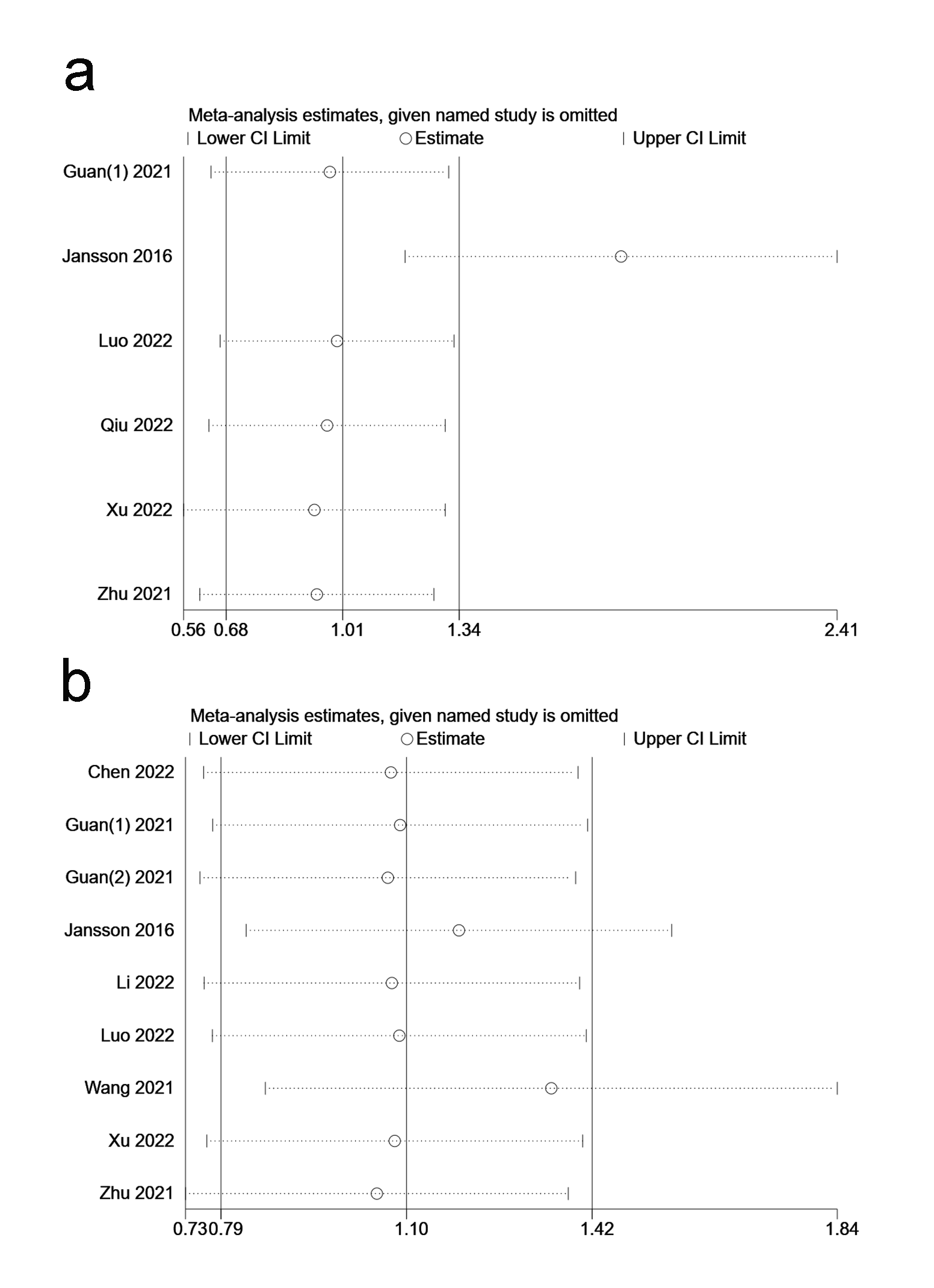


**Supplementary Figure 2.** Sensitivity analysis of OS for the pretherapy subgroup (a) based on sampling time and PFS/DFS/RFS/MFS for the posttherapy subgroup (b)


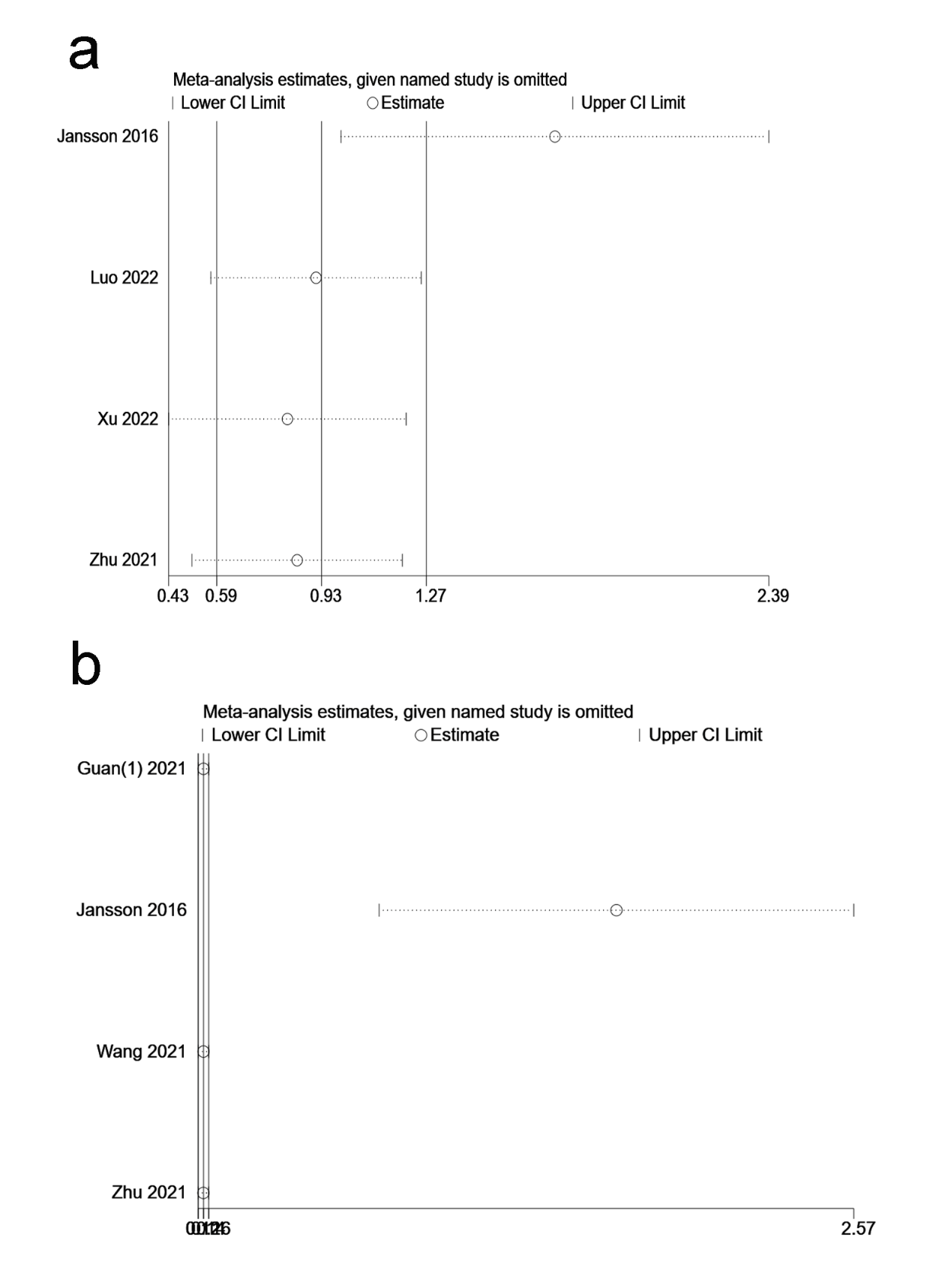

Supplement: Supplementary file 1 — Additional file 1: Supplementary Table 1. Search strategy. Supplementary Figure 1. Sensitivity analysis of OS (a) and PFS/DFS/RFS/MFS (b). Supplementary Figure 2. Sensitivity analysis of OS for the pretherapy subgroup (a) based on sampling time and PFS/DFS/RFS/MFS for the posttherapy subgroup (b). [file 12885_2023_11711_MOESM1_ESM.docx]
